# Results of Primary Treatment and Salvage Treatment in the Management of Patients with Non-Squamous Cell Malignant Tumors of the Sinonasal Region: Single Institution Experience

**DOI:** 10.3390/jcm12051953

**Published:** 2023-03-01

**Authors:** Urszula Kacorzyk, Marek Kentnowski, Cezary Szymczyk, Ewa Chmielik, Barbara Bobek-Billewicz, Krzysztof Składowski, Tomasz Wojciech Rutkowski

**Affiliations:** 1 I Radiation and Clinical Oncology Department, Maria Sklodowska-Curie National Research Institute of Oncology Gliwice Branch, Wybrzeże Armii Krajowej 15, 44-101 Gliwice, Poland; 2 Department of Oncological and Reconstructive Surgery, Maria Sklodowska-Curie National Research Institute of Oncology Gliwice Branch, Wybrzeże Armii Krajowej 15, 44-101 Gliwice, Poland; 3 Tumor Pathology Department, Maria Sklodowska-Curie National Research Institute of Oncology Gliwice Branch, Wybrzeże Armii Krajowej 15, 44-101 Gliwice, Poland; 4 Department of Radiodiagnostics, Maria Sklodowska-Curie National Research Institute of Oncology Gliwice Branch, Wybrzeże Armii Krajowej 15, 44-101 Gliwice, Poland

**Keywords:** sinonasal carcinoma, salvage, radiotherapy, surgery

## Abstract

Non-squamous cell carcinoma-related malignant sinonasal tract tumors (non-SCC MSTT) are rare and diverse malignancies. In this study, we report our experience in the management of this group of patients. The treatment outcome has been presented, involving both primary treatment and salvage approaches. Data from 61 patients treated radically due to non-SCC MSTT between 2000 and 2016 at the National Cancer Research Institute, Gliwice branch, were analyzed. The group consisted of the following pathological subtypes of MSTT: adenoid cystic carcinoma (ACC), undifferentiated sinonasal carcinoma (USC), sarcoma, olfactory neuroblastoma (ONB), adenocarcinoma, small cell neuroendocrine carcinoma (SNC), mucoepidermic carcinoma (MEC), and acinic cell carcinoma, which were found in nineteen (31%), seventeen (28%), seven (11.5%), seven (11.5%), five (8%), three (5%), two (3%) and one (2%) of patients, respectively. There were 28 (46%) males and 33 (54%) females at the median age of 51 years. Maxilla was the primary tumor localization followed by the nasal cavity and ethmoid sinus in thirty-one (51%), twenty (32.5%), and seven (11.5%) patients, respectively. In 46 (74%) patients, an advanced tumor stage (T3 or T4) was diagnosed. Primary nodal involvement (N) was found in three (5%) cases, and all patients underwent radical treatment. The combined treatment consisted of surgery and radiotherapy (RT) and was given to 52 (85%) patients. The probabilities of overall survival (OS), locoregional control (LRC), metastases-free survival (MFS), and disease-free survival (DFS) were assessed in pathological subtypes and grouped together, along with the ratio and effectiveness of salvage. Locoregional treatment failure was seen in 21 (34%) patients. Salvage treatment was performed in fifteen (71%) patients and was effective in nine (60%) cases. There was a significant difference in OS between patients who underwent salvage and those who did not (median: 40 months vs. 7 months, *p* = 0.01). In the group of patients who underwent salvage, OS was significantly longer when the procedure was effective (median: 80.5 months) than if it failed (median: 20.5 months), *p* < 0.0001. OS in patients after effective salvage was the same as in patients who were primary cured (median: 80.5 months vs. 88 months, *p* = 0.8). Distant metastases developed in ten (16%) patients. Five and ten year LRC, MFS, DFS, and OS were 69%, 83%, 60%, 70%, and 58%, 83%, 47%, 49%, respectively. The best treatment results were observed for patients with adenocarcinoma and sarcoma, while USC gave the poorest results in our set of patients. In this study, we indicate that salvage is possible in most patients with non-SCC MSTT with locoregional failure and that it may significantly prolong their overall survival.

## 1. Introduction and Aim of the Study 

Malignant sinonasal tract tumors (MSTT) are rare neoplasms that account for only 3% of head and neck carcinomas (HNC) and about 0.5% of all malignant diseases [1,2,3]. In contrast to other head and neck malignances, which are in the overwhelming majority squamous cell carcinomas, the pathology of MSTT is complex and diverse. Although the distribution of histological types varies in reported series, generally squamous cell carcinoma (SCC) accounts for about half of all MSTT and is followed by adenocarcinoma (10–27%), lymphoma (3–15%), adenoid cystic carcinoma (ACC), olfactory neuroblastoma (ONB), sarcoma, and mucosal melanoma, respectively, in 10%, 3%, 3%, and 2% [4,5,6,7,8,9,10,11]. Other, even more rare pathological types involve undifferentiated sinonasal carcinoma (USC), mucoepidermic carcinoma (MEC), small cell neuroendocrine carcinoma (SNC), and acinic cell carcinoma. The other reason for heterogeneity is a few tumor sites with various topography in the upper part of the head where MSTT may arise. Some correlation between tumor site and pathology type could be observed, like the fact that ethmoid tumors are mostly adenocarcinomas, or ONB, while SCC prevails in the maxillary sinus [5,8]. Latent and asymptomatic tumor growth or symptoms imitating sinusitis at the beginning of the disease usually turn into a late diagnosis and an advanced stage of the disease (stages T3–T4) when the tumor already infiltrates adjacent structures [3,9,12,13]. Numerous data indicate that less than 20% of primary-diagnosed MSTT are early-stage tumors [5,8,9,14,15,16,17]. Due to the above-mentioned rarity, heterogeneity, and challenging diagnosis, prospective studies on treatment efficacy have never been performed, and most treatment recommendations are based on one institution’s reports, usually with a limited number of cases. In this study, we report our experience with the management of patients with non-SCC MSTT. Treatment outcomes involving both primary treatment and a salvage approach are presented.

## 2. Material and Methods

A review of retrospective clinical data of 233 consecutive patients with MSTT treated between 2000 and 2016 at the National Cancer Research Institute, Gliwice branch, was performed. The study was conducted according to the guidelines of the Declaration of Helsinki and approved by the Ethics Committee of the Maria Skłodowska-Curie National Research Institute of Oncology, Gliwice Branch (decision code: KB/430-73/21; date of approval: 10 May 2021). As many as 81 patients underwent a palliative approach, and 12 patients with benign tumors were excluded. Additionally, 79 cases with SCC were excluded. Finally, the analyzed group consisted of 28 (46%) males and 33 (54%) females with a median age of 51 years. Thirty-six patients (59%) had never smoked, and 25 (41%) were smokers. The median duration of symptoms before diagnosis was 10 months. Maxilla was the primary tumor localization, followed by the nasal cavity and ethmoid sinus in 31 (51%), 20 (32.5%), and 7 (11.5%) patients, respectively. The 8th edition of the American Joint Committee on Cancer (AJCC) staging was used for pretreatment staging [18]. In 46 (74%) patients, an advanced tumor stage (T3 or T4) was diagnosed. Primary nodal involvement (N) was found in only 3 (5%) cases. The choice of the sequence of treatment methods depended mostly on the stage of the disease and tumor pathology. All patients underwent radical treatment. The combined treatment consisted of surgery and radiotherapy (RT) and was given to 52 (85%) patients. Among them postoperative RT alone was given to 43 patients, and RT combined with chemotherapy (chemotherapy—CHT, RT combined with CHT—CHRT) in 9 cases. RT alone was given to 2 patients. Induction chemotherapy was followed by RT alone in 2 patients and by CHRT in 3 patients. CHRT was given to 1 patient. Surgery alone was applied to one patient. All chemotherapy sessions were platinum-based. Monochemotherapy was used as concomitant therapy during RT. Platinum combined with either 5FU as PF or taxanes as TPF were used as induction agents. An RT dose in the range of 66–70 Gy was used to eradicate macroscopic tumor infiltration with RT alone or if surgery was R2. For eradication of microscopic extension of disease (surgery: R1) at least 66 Gy was used. For elective RT, the dose prescribed was in the range of 50–60 Gy.

Persistent disease was defined as either a local or regional tumor that did not disappear after treatment or recurred within 6 months of treatment completion. Recurrence was defined as either a local or regional tumor that recurred later than 6 months after treatment completion or that recurred anytime in patients who underwent postoperative treatment.

Salvage treatment was defined as an attempt to apply the radical management of a persistent tumor or recurrence after the completion of primary radical therapy. Successful (effective) salvage was reported when the treated tumor was either no longer observed for at least 3 months or remained stable for at least 6 months after the salvage procedure. Following the previous salvage, a subsequent recurrence was defined as either a recurrence or progression.

The analysis of the treatment outcome was based on follow-up data. Patients were seen 1–2 months after treatment completion, then every 3 months for the first year, every 6 months for another year, and then annually. At each follow-up visit, a physical examination, including palpation of the neck, was performed. Routine imaging was done with MRI, CT, or positron emission tomography scans every 6 months or at the physician’s discretion based on physical examination findings.

Both cumulative survival and tumor control rates were calculated using the Kaplan–Meier product-limited (actuarial) method. A *p* value of < 0.05 was considered statistically significant. A detailed analysis of the time and site of the primary treatment failure was performed. The ratio and effects of salvage were analyzed. The probabilities of overall survival (OS), locoregional control (LRC), metastases-free survival (MFS), and disease-free survival (DFS) were estimated from the end of primary treatment using the Kaplan–Meier product limit estimate and were compared using the log-rank test.

## 3. Results

### 3.1. Treatment Results in All Groups

**Figure 1:**
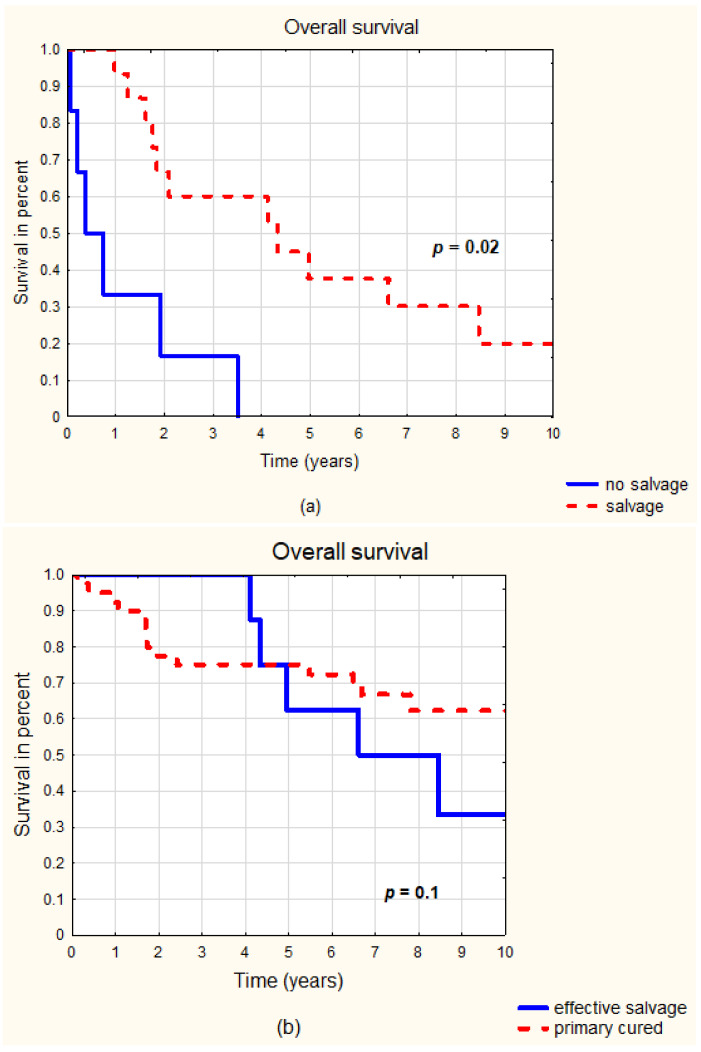
Overall survival considering salvage after primary treatment failure. (**a**) Patients with treatment failure: salvage vs. no salvage. (**b**) Patients cured primarily vs. cured after salvage.

The median follow-up in all groups was 86 months (range: 1–305 months). In general, locoregional treatment failure was seen in 21 (34%) patients. Persistent disease was found in four (6.5%) patients, and in all cases, it was localized in the primary site of the tumor. In one case, the persistent disease was concomitantly localized in the neck nodes. Recurrence appeared in 17 (28%) cases involving local, regional, or concomitantly locoregional sites in eleven, one, and five patients, respectively. The median time to recurrence was 21.5 months (range: 2–96 months). Distant metastases developed in ten (16%) patients, but only in six cases was this the sole reason for disease progression. The median time to metastases was 17 months (range: 3–60 months). In the remaining four patients, metastases appeared in patients with persistent disease (one case) or in patients with recurrence (three cases). Five and ten years LRC, MFS, DFS, and OS were 69%, 83%, 60%, 70% and 58%, 83%, 47%, 49%, respectively. Despite locoregional failure, six (29%) patients have not been admitted to salvage procedures due to: (a) quickly progressing persistent tumor after primary treatment (two cases); (b) advanced stage of recurrent disease (two cases); (c) lack of pathologically proven recurrence despite radiological progression (one case); and (d) persistent tumor without progression for about 7 years. Salvage treatment was given to the remaining fifteen (71%) patients and was effective in nine (60%) cases. In the group with effective salvage, surgery was undertaken as the first treatment modality in eight patients, and in one patient, it was RT. In five patients from the group, recurrence occurred more than once. In these cases, the salvage approach (surgery or RT) was given from two to five times. There was a significant difference in OS between patients who underwent salvage and those who did not (median: 40 months vs. 7 months, *p* = 0.01). In the group of patients who underwent salvage, OS was significantly longer when the procedure was effective (median: 80.5 months) than if it failed (median: 20.5 months), *p* < 0.0001. What is interesting is that OS in patients after effective salvage was the same as in patients who were primary cured (median: 80.5 months vs. 88 months, *p* = 0.8). (Figure 1). A detailed distribution of patients’ characteristics according to individual pathologies is presented in Table 1. The results of primary treatment, the salvage ratio, and its effects distributed according to pathological type and in all groups are presented in Table 2.

### 3.2. Adenoid Cystic Carcinoma

Our group consisted of 19 (31%) patients with ACC. The maxillary sinus (68%) and nasal cavity (21%) were the most common sites of primary ACC tumors. The median age in this group was 56 years, and the women/men ratio was 13/6. In all but one patient, surgery was the primary treatment, which was followed by RT. Only one patient did not receive postoperative RT due to the lack of patient agreement. Additionally, due to an unresectable primary tumor, one patient received RT alone following induction CHT. The median time between surgery and RT was 4 months. In 10 cases 66–70 Gy was administered due to a residual macroscopic tumor. In the remaining eight patients 57.6–60 Gy was administered to eradicate the microscopic disease. In seven (37%) cases, local recurrence of the disease was found at the median time of 43 months (range: 2–96 months) after primary treatment completion. In one case from this group, 5 months after a local recurrence, a regional one appeared, and in the next 10 months, a distant spread developed. Distant metastases appeared in three more patients from this group in a median time of 35 months. There were also three other primary malignant tumors that appeared in patients 25, 7, and 209 months after primary treatment completion. Salvage was provided to four (57%) of the seven patients with locoregional failure. In three patients, it was surgery for the first failure and RT for the subsequent ones. In one case, RT alone was provided. In two patients, salvage was 50% effective. Five and ten years LRC, MFS, DFS, and OS were 77%, 82%, 62%, 74% and 46%, 82%, 30%, 38%, respectively.

## 4. Undifferentiated Sinonasal Carcinoma

Our group consists of 17 (28%) patients with USC pathology. The nasal cavity and maxillary sinus were the most common sites of primary tumors in this group, accounting for 41% each. The median age was 55 years, with a female/male ratio of 6/11. Additionally, in this group, in most cases, primary surgery followed by RT/CHRT was the main approach and was carried out in 14 (82%) patients. In three patients from this group CHRT was used. In three patients, no surgery was performed due to the advanced stage of the disease. In two patients from this group, induction chemotherapy was given with either RT or CHRT, and in one case, CHRT alone was given. The median time between surgery and RT was 2.5 months. After treatment completion, persistent tumors were found in two cases. In five patients recurrence appeared in a median time of 16 months. From this group, in three cases, local recurrence alone was found. In the next two patients, there was also nodal recurrence that preceded local recurrence in one case and followed it in the second one. Metastatic disease was found in four cases, but only in one patient was it the only reason for failure. Out of seven patients with locoregional failure, salvage was provided to six (85.5%). Surgery was used on three patients, and on the three others, RT was used as salvage. In two (33%) patients, salvage was effective; both cases had surgery. Five and 10 years LRC, MFS, DFS, and OS were 55%, 78%, 51%, 64% and 55%, 78%, 51%, 25%, respectively.

### 4.1. Sarcoma

Our group consisted of seven (11.5%) patients with sarcoma. The nasal cavity and maxillary sinus were the most common sites of the primary tumor in this group, accounting for 43% each. The median age in this group was 44 years, and the man/woman ratio was 3/4. In all but one patient, surgery was the primary treatment, which was followed by RT. In one patient, RT alone was applied due to an advanced tumor, and RT was not completed because the patient was lost to therapy after the dose of 28 Gy. Additionally, in one patient, postoperative RT was preceded with induction chemotherapy due to progression shortly after surgery. The median time between surgery and RT was 3 months. In one patient from this group, treatment failure was found as a persistent tumor. Salvage surgery was performed, followed by RT, but only the subsequent salvage and stereotactic RT were successful, and the patient was eventually cured. Five and 10 years LRC, MFS, DFS, and OS were 83%, 83%, 68%, 71% and 83%, 83%, 68%, 71%, respectively.

### 4.2. Olfactory Neuroblastoma

There were seven (11.5%) patients with ONB in our group. The ethmoid sinus (57%) and nasal cavity (43%) were the most common sites of the primary tumor in this group. The median age was 39 years, and the male/female ratio was 4/3 in this series. Additionally, in this group, in most cases, primary surgery followed by RT was the main approach and was carried out on five patients. In two patients, no surgery was performed due to the advanced stage. Concomitant CHRT was used in one patient from this group, and RT alone was used in another. The median time between surgery and RT was 2.8 months. In five cases, 60 Gy was given to eradicate microscopic diseases. In two cases, 66–68 Gy were given due to the macroscopic tumor. There were four treatment failures: a residual tumor in one case and recurrence in three cases. A patient who had a persistent tumor had a distant spread of the disease. Salvage was given to two (50%) patients from this group, and in both cases, it consisted of surgery, RT, and systemic treatment repeated a few times. This effort was successful for both patients. Five and 10 years LRC, MFS, DFS, and OS were 57%, 85%, 57%, 71% and 43%, 85%, 43%, 57%, respectively.

### 4.3. Adenocarcinoma

Our group consisted of five (8%) patients with this pathology. The maxillary sinus (80%) and nasal cavity (20%) were the most common primary sites of this malignancy. The median age was 56.6 years, and the male/female ratio was 2/3. In all cases, primary surgery followed by RT was the main treatment approach. The median time between surgery and RT was 2.3 months. In four cases, 60 Gy was given to eradicate the microscopic disease. In one case, 66 Gy was given due to a residual macroscopic tumor. The results of primary treatment were excellent; in only one patient, nodal recurrence appeared 21 months after primary treatment completion. Surgical salvage was successful in this case. Five and ten years LRC, MFS, DFS, and OS were 80%, 100%, 80%, 100% and 80%, 100%, 80%, 100%, respectively.

### 4.4. Small Cell Neuroendocrine Carcinoma

There were three (5%) patients with a small cell neuroendocrine carcinoma in this group. It was localized in the nasal cavity and maxillary sinus in two and one case, respectively. There were two women and one man, with a median age of 53 years. In two patients, primary surgery followed by RT was carried out, and in another patient, CHRT was used. The median time between surgery and RT was 3.2 months. All patients were cured, and only in one case did a distant spread to the lung appear about a year after the completion of primary treatment. CHT was not effective, and the patient died about 2 months later.

### 4.5. Mucoepidermic Carcinoma

There were two (3%) patients with mucoepidermic carcinoma in this group, and both cases had a primary tumor localized in the maxillary sinus. They were a man and a woman, ages 30 and 63, respectively. In both patients’ surgeries, primary treatment was followed by RT with elective doses. The median time between surgery and RT was 2.9 months. Both patients were cured, but one of them died 21 months later due to another reason.

### 4.6. Acinic Cell Carcinoma

There was one (2%) patient with this rare pathology in our group. The tumor in the 59-year-old woman was localized in the nasal cavity. The patient underwent surgery, which was followed by RT 2.5 months after surgery at a dose of 60 Gy. After 4 years of follow-up, a local recurrence was found, and the patient underwent stereotactic RT. After the next 4 years, the patient underwent endoscopic surgery due to a subsequent recurrence. There was another stereotactic RT in the next 4 years due to the next recurrence and four cycles of palliative CHT in the next 2 years due to a subsequent recurrence. The patient died in the next 1.5 years due to the progression of local infiltration of the cancer.

## 5. Discussion

The rarity of MSTT, which is even more sparse due to diverse pathology, means that reports considering this type of cancer are usually from one institution and usually with a limited number of cases. In this study, we described the results of the radical treatment of 61 patients who suffered from MSTT, taking into account the follow-up period and the results of a salvage approach. To refer to as many clinical outcomes as possible, we presented the results of an entire group first and subsequently the results in each pathological cohort. All patients were treated at a single cancer center, the National Cancer Research Institute, Gliwice branch, Poland. The patient’s distribution was generally consistent with other series, with the majority of patients having advanced stages (T3, T4), and only 5% of them having involved regional lymph nodes [19,20]. There were more women in our group, probably due to a relatively large subgroup of patients with ACC and USC. Females dominating in these groups were also found in other series [21,22]. The predominating primary site of the MSTT differs slightly between our cohort and other reported groups. We found most cases with primary infiltration in the maxilla, followed by the nasal cavity, whereas Hafstrom et al. reported an inverse distribution, and Dirix et al. pointed to the ethmoid sinus as the most frequent primary site of MSTT [20,21]. Contrary to other authors’ reports listing adenocarcinoma as the second most common malignancy in this region [23], ACC and USC were predominating in our group. 

A five year OS rate of 70% established in our group is comparable to the survival rate reported by other authors (38–69%) [6,7,14,21,24,25,26,27,28]. Due to the relatively long follow-up period, we were also able to assess the 10 year OS, which was 49%. Other authors reported a 10 year OS rate in the range of 35–48% [21,24,25].

Five and 10 years of DFS were at 60% and 47% in our group, which is comparable to 42–63% and 54–59%, respectively, reported by other authors [21,24,25]. In our group, only four (6.5%) patients were never in remission after primary treatment. The ratio of persistent disease after primary treatment was between 6% and 14%, which was also reported by other authors [21,28]. In patients with a persistent tumor, salvage was performed in two patients (50%) and was finally successful in both cases. Mirghani et al. reported effective salvage in only two patients (9%) out of twenty-two with a persistent tumor after primary treatment [28].

In our group, locoregional failure was seen in 38% of cases but isolated local ones in 18%. In one of the largest series aiming to report recurrences during follow-up in MSTT patients performed by Zochi et al., at least one recurrence during follow-up was shown in 28% of patients, and in almost 75% it developed in the first three years after primary treatment [29]. In that study, the median time to first relapse was 17 months [29]. Mattaveli et al. assessed the median time to recurrence as 18 months, which is shorter than the 21 months assessed in our study [25]. According to Mattavelli et al., over 60% of all recurrences in the group were local, which is similar to our group, where 65% of patients with recurrences developed it in a primary site [25]. Additionally, other authors show local recurrence as the most common site of relapse in MSTT patients in the range of 15–73% and an average of 30–40% [1,7,14,19,20,25,28,30,31,32]. However, the most commonly cited factors increasing the risk of local recurrence, such as T-staging and pathology, as well as primary treatment performed outside referral centers, could also be an adverse factor [1,25,29]. Due to the high risk of local failure, post-treatment surveillance seems essential for early detection of failure. It is, however, challenging due to lack of both surveillance recommendations (how often and what diagnostic procedure is optimal) and evidence of its influence on survival prolongation [33]. Despite other benefits of surveillance like comprehensive nutritional assessment, rehabilitation after surgery, and RT [10], the evidence of an effective salvage may support the significance of early detection of treatment failure [34]. Most of the data concerning the role of salvage for MSTT patients refers to SCC as the most common pathology. For this malignancy, salvage is possible in 30–70% of patients [34,35,36]. It has been shown that even patients in whom salvage was not effective presented an improved OS compared with those with failure but no salvage at all. Moreover, effective salvage appeared to compensate for failure, giving the same ultimate OS as in primarily cured patients [34]. In non-SCC patients, the relevance of salvage is more difficult to assess due to the pathological diversity in this group. For such patients, Kaplan et al. proposed a therapeutic algorithm that considered, among other things, pathology and the site of recurrence. Based on the series of 49 patients with recurrence, surgery was strongly recommended for low-grade tumors, while a rather palliative approach was recommended for high-grade lesions with orbital or skull-base invasions [2]. Mattaveli et al. also suggested a multiparametrical score defining groups A, B, and C with excellent survival estimation, intermediate prognosis, and poor estimated survival comparable to those of metastatic head and neck cancer, respectively. The authors concluded that, similarly to primary tumors, in the recurrent setting, histology and tumor biology are critical, strongly influencing final results. In cases of unfavorable pathology, SNC has been included, while ONB and USC presented the best survival estimates in this analysis [25]. Contrary to this observation, USC malignancies gave the poorest results in our set of patients, with the highest ratio of recurrence and relatively low salvage success. Additionally, according to other authors, the prognosis of patients with USC remains poor [21,37]. We obtained the best primary treatment results for patients with adenocarcinoma and sarcoma. Despite a relatively higher ratio of locoregional failure, due to effective salvage, patients with ACC and ENB could be considered good predictors. Additionally, according to Hafstrom et al., adenocarcinoma and ENB have a relatively good prognosis [21].

Not much data concerning the results of salvage for patients with the presented pathological types are available. Mirghani et al. found salvage to be an effective treatment in 20% of isolated local recurrences and in 16% of cases with both local and nodal failure [28]. In our series, time to recurrence did not significantly influence salvage effectiveness and was similar in those who experienced effective or ineffective salvage. Our data suggest that salvage was possible in over 70% of recurrent patients and was effective in 60% of those who underwent this procedure. The results of salvage assessed in this group of patients are consistent with those obtained for SCC patients [34]. We were able to confirm that salvage is an effective procedure and may significantly prolong OS, reducing the adverse effect of recurrence for patients with non-SCC MSTT.

Isolated regional relapse usually is rare. In most series, it does not exceed 10%, usually being in the range of 4–6%. We found recurrent disease in regional nodes in 4% of our patients. Mirghani et al. described the issue of regional failure in detail, and pointed out that nodal recurrence appeared in 10% of patients in all groups, but 6.5% while considering patients without local failure, and 4% of those with initially cN0 [28]. Despite generally rare regional relapse pathological types like SCC or USC without prophylactic neck treatment may develop more nodal recurrences than ACC or MEC [38]. Other risk factors include T-stage in the context of local invasiveness, especially at sites with a rich lymphatic network [20,39,40]. Recommendations for elective nodal treatment to prevent regional recurrence are not well defined and vary between authors, usually due to the heterogeneity of the groups and a limited number of neck relapses [28]. For patients with an isolated nodal recurrence, salvage remains a good option. In our group, only one patient presented with an isolated nodal relapse and underwent effective salvage. In two patients in whom a nodal recurrence developed prior to or simultaneously with a local one, surgical salvage was effective in both cases. In the remaining three cases, local recurrences appeared prior to a nodal one. The median preceding time was 5 months, which may suggest a rather nodal progression from local recurrence than metastatic failure after primary treatment. Such a scenario has also been suggested by Mirghani et al., who stressed the separation between isolated neck recurrence and that associated with local failure. Such misinterpretation may lead, according to this author, to an overstated indication for prophylactic neck management [28]. In addition, successful nodal salvage has been reported by other authors. Cantu et al., in a group of 399 patients with maxillary sinus cancer, found 281 recurrences and 31 isolated nodal ones among them. Due to effective salvage, only two of them died of nodal-only metastases [41]. Dirix et al. found an isolated nodal recurrence in six patients in a group of one hundred and twenty-seven patients with MTSS. All of them underwent salvage neck dissection, followed by postoperative RT (no elective RT was given during primary treatment), which was effective, and none of them died due to nodal relapse [20].

### 5.1. Adenoid Cystic Carcinoma

ACC is a relatively slow-growing tumor characterized by perineural invasion and a high rate of local recurrence. In the distribution of histological types of MSTT, ACC usually accounts for about 10% [8], but in our series, it was the most common type (SCC was excluded from this analysis). In our group, females were dominant (sex ratio: 2.0). Atallach et al. also found more females in their group (sex ratio: 1.5) [42]. The five to ten years local recurrence rate is 30% to 75% [43]. In our group, locoregional recurrence was found in 37% of patients, and in 57% of them, salvage was performed. It was successful in 50% of patients who underwent this procedure. It resulted in a 5 and 10 years OS ratio of 74% and 38%, respectively. Other authors report 5 and 10 years of OS at 68–85% [42,44] and 52–67%, respectively [42,44,45]. Long-term survival in ACC is usually affected by a high risk of distant metastases (40–50%), but we found it only in 15% [42,44].

### 5.2. Undifferentiated Sinonasal Carcinoma

Sinonasal undifferentiated carcinoma is a rare and aggressive tumor. This malignancy was second as to the number of subgroup cases in our study, with almost two times more females in the group, which is in contrast with other data [46]. Over 80% of our patients undergo surgery followed by RT or CHRT. Kuo et al. showed that surgery, RT, and CHT as a combined modality are the most effective treatment, with a 5 year survival rate of 41.5% [47]. Other authors showed 5 years of OS after surgery combined with RT in the range of 36–39%, indicating that RT is a critical component in the treatment [46]. Additionally, CHT is almost always included as part of the therapy regimen, and the role of induction CHT has been raised [48]. In general, the overall 5 year survival rate for this malignancy is less favorable than for other MSTT malignancies. It was also confirmed in our results. Despite that, locoregional failure was found in 41% of our patients, and salvage was performed in all but one case. It was effective in one-third of patients.

### 5.3. Sarcoma

Sarcomas are extremely rare, accounting for only ~1% of all the malignancies in the head and neck region. Moreover, the nasal cavity and paranasal sinus location represents only about 5% of all head and neck sarcomas. Malignant peripheral nerve sheath tumor (MPNST) was diagnosed in three cases in our group, while in the literature, not more than 25 cases have been reported so far [49]. In our group, five out of seven cases underwent surgery followed by RT. Such management seems to be optimal for these type of malignances. Although preoperative RT is well tolerated and provides a high rate of local control [50], RT is most commonly followed by definitive surgery [51]. Treatment results in our group were good and 5 and 10 years OS was 71%. In one case, recurrence salvage was performed and appeared to be effective. Five years of OS and the local control ratio reported by other authors are in the range of 56–82% and 41–83%, respectively [50,52]. One should remember, however, that pathological subtype, site of primary tumor, histological grade, and percentage of gross total resections, among others, may significantly impact the outcome.

### 5.4. Olfactory Neuroblastoma

Olfactory neuroblastoma is a rare tumor arising from the olfactory neuroepithelium in the sinonasal cavity. ENB presents a bimodal age distribution with peaks in the second and sixth decades [10]. It was the youngest subgroup in our study, with a median age of 39 years. In general, the combination of surgery and RT is the most frequently used treatment and was associated with the best average survival results (65%) in the meta-analysis performed by Dulguerov et al. The 5 year and 10 year OS rates in our group were 71% and 57%, respectively, which is better than in other reports. The mean overall survival and disease-free survival at 5 years was 45% (range: 0–86%), and the average OS at 10 years was 52% [53]. Despite the main roles of surgery and RT, CHT has been increasingly used by Thawani [10]. There was a local failure of 57% in our group, which is higher than the 29% reported by Dulguerov et al. Salvage was possible in 50% of our patients, which is consistent with others, indicating possible salvage in 33–50% of patients with a local recurrence [54]. A salvage approach in our group was multimodal according to subsequent recurrences in these patients and turned into additional years of overall survival.

### 5.5. Adenocarcinoma

Contrary to other data, which indicate this type as the second most common malignancy, we found it only in 8% in our group (Castelnuovo and Arnold) [23,24]. We found the maxilla most often as the primary site of this tumor, while others pointed out the nasal cavity and ethmoid sinus (Bhayani) [54]. We noticed excellent results from surgery followed by RT. A successful salvage neck dissection due to reginal recurrence was performed, resulting in a 5 years OS of 100%. Arnold et al. in a group of 21 cases reported 53% of 5 and 10 years OS. (Arnold) [24]. In fact, the 5 year overall survival (OS) rates in this group vary widely among studies, ranging from 36% to 86% (Maccariello) [55].

### 5.6. Small Cell Neuroendocrine Carcinoma

A rare cancer arising mostly in the ethmoid sinus [56]. There were three patients with this tumor in our group, and the primary localization was the maxilla in two of them. According to the literature, surgery followed by RT remains the main treatment approach, although some data indicate that adding CHT as an induction or as CHRT after surgery may improve treatment results [57,58]. Three of our patients underwent surgery followed by RT, and CHRT was given to one patient. The main reason for the failure of this malignancy is the local recurrence or distant spread of the disease [59]. Although all our patients were locoregionally cured, one of them died due to distant metastases.

### 5.7. Sinonasal Mucoepidermoid Carcinoma

Mucoepidermoid carcinoma is a common salivary gland malignancy that rarely arises in the sinonasal region. This malignancy was found in only 3% of the patients in our group. Maxilla is the most typical primary site for this tumor [60], and all our cases were localized in maxilla. Generally, treatment includes surgery followed by RT. Trantafilou et al. reported results of treatment for 164 patients with 1, 2, and 5 years of OS of 83%, 77.0%, and 57%, respectively [60]. Such therapy was effective for our patients, and none of them experienced treatment failure.

### 5.8. Acinic Cell Carcinoma

Acinic cell carcinoma is a rare cancer of this region. A review of the National Cancer Database reported 28 such patients treated between 2004 and 2016. Most of these tumors arose in the nasal cavity [61], and this was also the primary site of the tumor in our patient. Biron et al. pointed out that all 18 of his cases were low-grade [62]. Surgery alone was the main treatment option in a cohort described by Khirsagar et al. [61]. Overall survival at 1, 5, and 10 years was 100%, 84.3%, and 52.3–72.2%, respectively [61,62]. A meta-analysis of survival from cases in the literature performed by Biron et al. estimated 10 year recurrence-free survival at 92.9% [62]. Our patient presented with 14 years of OS, despite local recurrences for the last 10 years that had been treated subsequently. A good result from a few salvage attempts was probably due to the low grade of this tumor. Our result confirms that it is a rare entity with relatively good long-term outcomes, and salvage may be effective [61,62].

Patients suffering from MSTT require a multidisciplinary team approach not only at diagnosis but also during follow-up. Multidisciplinary care of patients with their survivorship issues is needed including rehabilitation or comprehensive nutritional assessment. Of special importance is support in the management of the consequences of surgery or RT, including the prevention of delayed radiation-induced complications (second malignancies, hypothyroidism, and tissue necrosis). Moreover, surveillance is also significant because it helps facilitate an early diagnosis of recurrence. Our data indicates that salvage is effective, but often a multimodal and multidisciplinary team should decide what salvage option is optimal for a particular patient in an individual clinical situation, taking into account stereotactic radiosurgery and intensity-modulated particle therapy (i.e., protons and ^12^C-carbon ions) [16,63]. Due to the rarity and heterogeneity of MSTT cancer registries, international clinical studies dedicated to patients with MSTT could be proposed as a solution for this rare disease [64].

This study has several limitations common to retrospective studies, which are even more pronounced here due to the diverse pathology and low number of cases. On the other hand, even a few cases of a rare pathology well described in a clinical scenario could be beneficial. We believe that our data will support general knowledge about this disease and may add value to the discussion about the management of patients with MSTT in the future. 

## 6. Conclusions

Patients with non-SCC MSTT present a diverse prognosis that is related to several clinical and tumor-related factors. In most cases, a multimodal primary treatment is suggested to decrease the risk of local recurrence, which is the main reason for failure. Recommendations are, however, sparse due to the rarity of such malignancies and the almost complete absence of clinical trials. In this study, we indicate that salvage is possible in most patients with non-SCC MSTT with locoregional failure and may significantly prolong their overall survival.

## Figures and Tables

**Table 1:** Patients’ characteristics according to histopathological types and in all groups. ACC—adenoid-cystic carcinoma, USC—undifferentiated sinonasal carcinoma, ENB—olfactory neuroblastoma, MEC—mucoepidermic carcinoma, and SNC—small cell neuroendocrine carcinoma. CHRT—radiochemotherapy.

Pathology	No/%	Age (Median)	Man/ Woman	Maxilla	Nasal Cavity	Ethmoid Sinus	Other	Surgery + pRT/CHRT	Surgery Alone	RT/CHRT Alone	RT Dose (Gy)
											66–70/50–60
ACC	19/31	52	6/13	13	4	1	orbit	17/1	1	1/0	10/8
USC	17/28	54	6/11	7	7	2	frontal sin.	11/3	0	0/3	8/9
Sarcoma	7/11.5	44	3/4	3	3	0	frontal sin.	6/0	0	1/0	2/5
ENB	7/11.5	39	4/3	0	3	4	-	4/1	0	2/0	2/5
Adenocarcinoma	5/8	56.5	2/3	4	1	0	-	5/0	0	0/0	1/4
SNC	3/5	53	1/2	2	1	0	-	1/1	0	0/1	2 /1
MEC	2/3	46.5	1/1	2	0	0	-	1/1	0	0/0	0/2
Acinic cell carcinoma	1/2	59	0/1	0	1	0	-	1/0	0	0	0/1
All groups	61/100	51	23/38	31	20	7	3	46/7	1	4/4	25/35

**Table 2:** Primary treatment results and results of salvage according to histopathological types and in all groups. Rec—recurrence, L—local, N—nodal, LN—local and nodal, ND—no data, ACC—adenoid-cystic carcinoma, USC—undifferentiated sinonasal carcinoma, ENB—olfactory neuroblastoma, MEC—mucoepidermic carcinoma, LRC—locoregional control, SNC—small cell neuroendocrine carcinoma. MFS—metastases-free survival, OS—overall survival.

Pathology	No/%	Persistant Tumor	Rec			Rec %	Met %	5 Year LRC (%)	5 Year MFS (%)	5 Year OS (%)	10 Year OS (%)	Salvage No/%	Effective Salvage No/%
			L	N	LN								
ACC	19/31	0	6	0	1	37	26	77	82	74	38	4/57	2/50
USC	17/28	2	3	0	2	41	23.5	55	78	64	25	6/88	2/33
Sarcoma	7/11.5	1	0	0	0	14	0	83	83	71	71	1/100	1/100
ENB	7/11.5	1	1	0	2	28	14	57	85	71	57	2/50	2/100
Adenocarcinoma	5/8	0	0	1	0	20	0	80	100	100	100	1/100	1/100
SNC	3/5	0	0	0	0	0	33	100	63	33	33	0/0	0/0
MEC	2/3	0	0	0	0	0	0	100	100	50	50	0/0	0/0
Acinic cell carcinoma	1/2	0	1	0	0	100	0	0	100	100	100	1/100	1/100
All groups	61/100	4	11	1	5	31	16	69	83	70	49	15/24.5	9/60

## Data Availability

The data are available upon request from the corresponding author.
